# Editorial: The Psychology of Sport, Performance and Ethics

**DOI:** 10.3389/fpsyg.2021.658457

**Published:** 2021-03-05

**Authors:** Yair Galily, Roy D. Samuel, Edson Filho, Gershon Tenenbaum

**Affiliations:** ^1^Interdisciplinary Center Herzliya, Herzliya, Israel; ^2^Wheelock College of Education and Human Development, Boston University, Boston, MA, United States

**Keywords:** ethics, exercise, coronavirus, sports, athlete, coaches

Sport and exercise are considered a significant factor in the lives of many people (see Simon, 2018; Galily, 2019, 2021). While many people exercise and are engaged in several physical activities, others view and watch sport competitions and events. Indeed, evenw those who are unconcerned with sporting games, and are critical of athletic rivalry, are often affected by them due to their relationships with other enthusiasts, and the impact of sport on human language, thought, and culture. The current limitations posed on exercise engagement and sport participation due to the COVID-19 global pandemic further emphasized the importance of exercise and sport for physical and mental health, and interpersonal interactions (Samuel et al., 2020).

Because sport is a meaningful form of social activity that affects politics, economy, and behavioral norms, it brings up a wide range of issues, some of which attract research and scientific inquiry (Simon, 2018: p. 3). While research in various domains of sport pertain to contextual and social factors, some were devoted to the factors which promote or hinder dishonesty. In addition, advances in technology and the growth of social media usage, along with substantial financial investment in sport, raise significant ethical questions worth elaboration and inquiry.

The aim of the current compendium on the psychology of sport, performance, and ethics was to assemble both theoretical and applied research from experts within the field of sport psychology, sociology, performance, and exercise. Twelve articles, written by researchers from Brazil, China, Germany, Israel, the Netherlands, Norway, Portugal, Spain, Taiwan, and the United States, were divided into four chapters. The first chapter, “Decision-Making Challenges in Dynamic Sporting Environments” holds four articles: Gershgoren et al. introduce the chapter *Perceived Performance in Team Sports Questionnaire* to capture the team members' perception of their team's performance. Samuel et al.'s case study adopted an intrinsic mixed-methods methodology to investigate the implementation of the video assistant (soccer) referee system within the Israeli Premier League context. Johansen and Erikstad investigated elite referees' positioning in the field of play (distance, angle, and insight) when making correct and erroneous decisions in potential penalty situations. Finally, Del Campo and Martin assessed the effects of manipulating video speeds on visual behavior and decision accuracy of 10 amateur football assistant referees when watching video sequences of 24 possible offside actions.

The second chapter, entitled “Integrity and Ethical Issues in Sport Psychology,” comprises three articles: Englert and Schweizer tested the capability of individuals to judge correctly whether athletes are lying or telling the truth, and suggested that participants can distinguish between true and false statements, but only for some clips and not for others; indicating that some players were better at deceiving than others. Tamir and Bar-eli argued that despite fierce objections and extensive criticism, the video assistant referee system represents an important turning point in modern professional soccer, and moreover, it accomplishes a moral revolution in the evolution of the sport domain. The newly developed technologies enhanced the sport's professional standards and its public image and prestige, and especially the moral standard of fair play. Lastly, to examine effective communication and coordination within the context of referee teams, Sinval et al. developed the Referee Shared Mental Models Measure (RSMMM).

The third chapter, “Performance Enhancement and Aggressive Behavior” congregates three issues. Wang et al. offered support for the meshed control theory and indicated the dynamic nature of neuromotor processes for the superior performance of athletes in challenging situations. Strenge et al. outlined how the domain of cognitive interaction technology research addresses ethical issues and presented an empirical study in the context of a new measurement and assessment system for training in karate. In two experiments, Geng et al. explored the implicit link between the color red and aggressiveness as well as the color blue and agreeableness in students and in Taekwondo athletes.

The fourth chapter labeled “Parental Style and Involvement in Sport,” includes two studies: Yaffe et al. tested the relationships between sport type (team or individual) and parenting styles (authoritative vs. non-authoritative), and moral decision-making in sport and sport values. Correspondingly, Lev et al. explored the nature of parental involvement in youth basketball in Israel with regards to parenting style and in the context of dilemmas and ethical issues.

In line with Sly et al.'s (2020) notion, our practice is increasingly characterized by a complex interaction among clientele, roles, services, and competencies. Hence, it is our hope that insights of this diverse collection of articles further expands the boundaries of issues related to athletes and coaches and the whole sporting eco-system. Acknowledgment of the recent diversification of sport psychology provision has been evidenced by Division 47 (Sport, Exercise, and Performance Psychology; SEP) of the American Psychological Association, advocating that SEP psychology must be conceptualized as a subdiscipline of performance psychology; that is, a domain of study and practice concerned with the identification, development, and execution of skills and abilities required to achieve excellence within a series of diverse performance domains, such as business, military, health care, education, and the performing arts (Sly et al., 2020: p. 87). In our humble effort to create a more intelligible picture of what characterizes and bounds our discipline, we conclude by saying: Let the games (and reading) begin!

## Author Contributions

All authors listed have made a substantial, direct and intellectual contribution to the work, and approved it for publication.

## Conflict of Interest

The authors declare that the research was conducted in the absence of any commercial or financial relationships that could be construed as a potential conflict of interest.
